# Comparison of the Areas Under the Curve of Vancomycin Continuous vs. Intermittent Infusion in Critically Ill Pediatrics: A Randomized Clinical Trial

**DOI:** 10.5812/ijpr-145933

**Published:** 2024-04-28

**Authors:** Baran Roshan N.S., Bahador Mirrahimi, Farhad Najmeddin, Seyedeh Narjes Ahmadizadeh, Azita Behzad, Seyedeh Masumeh Hashemi, Maryam Alemzadeh, Niloufar Taherpour

**Affiliations:** 1 Department of Clinical Pharmacy, School of Pharmacy, Shahid Beheshti University of Medical Sciences (SBMU), Tehran, Iran; 2 Department of Clinical Pharmacy, Faculty of Pharmacy, Tehran University of Medical Sciences (TUMS), Tehran, Iran; 3 Department of Pediatric Intensive Care, Mofid Children Hospital, Shahid Beheshti University of Medical Sciences (SBMU), Tehran, Iran; 4 Prevention of Cardiovascular Disease Research Center, Shahid Beheshti University of Medical Sciences (SBMU), Tehran, Iran

**Keywords:** Vancomycin, Drug Monitoring, Administration and Dosage, Critical Care, Pediatrics

## Abstract

**Background:**

Providing data on the superior efficacy of vancomycin administered based on the area under the curve over 24 hours to the minimum inhibitory concentration of vancomycin (AUC_24_/MIC) is crucial. However, data on dosing and monitoring of vancomycin pharmacokinetics in the pediatric population are limited. Previous findings have showed that intermittent infusion of vancomycin (IIV) may not achieve the desired levels, continous infusions of vancomycin (CIV) reach the desired serum concentration faster than IIV and are associated with reduced nephrotoxicity.

**Objectives:**

This study aimed to compare the serum concentrations, AUC_24_, clinical variables, and adverse effects of two vancomycin administration methods in the pediatric population.

**Methods:**

This study was a double-blind, randomized, controlled clinical trial conducted at a tertiary children's teaching hospital. Inclusion criteria were age between 2 months and 15 years and weight less than 67 kilograms, with exclusion criteria including renal impairment. Participants were divided into CIV and IIV groups following distinct administration protocols. Demographic, clinical, and laboratory data, including vancomycin serum concentrations, were compiled. Assessments included pediatric mortality risk, pediatric sequential organ failure assessment, and regular temperature monitoring. Pharmacokinetic analysis was conducted using Monolix software 2023R1. Primary endpoints were vancomycin serum levels and AUC_24_ between cohorts on day three, with nephrotoxicity and additional adverse drug responses evaluated.

**Results:**

Sixty-eight patients in the pediatric intensive care unit (PICU) were allocated to either CIV (33) or IIV (35) for vancomycin treatment. In the CIV group, 82% of patients achieved an AUC_24_ ≥ 400 mg.h/L, compared to 23% in the IIV group. Continuous infusions of vancomycin demonstrated a greater AUC_24_ (587.7 ± 184.4 mg.h/L vs. 361.9 ± 113.2 mg.h/L, P < 0.05) compared to IIV. Two cases of nephrotoxicity were reported, one in each group, with mortality and adverse events being comparable between the two groups.

**Conclusions:**

This study demonstrated that continuous vancomycin infusion has a higher success rate in safely achieving therapeutic vancomycin levels in PICU patients compared to intermittent vancomycin infusion.

## 1. Background

Vancomycin, an antibiotic of the glycopeptide class, is effective against gram-positive bacteria (1, 2). It is considered the treatment of choice for infections caused by methicillin-resistant *Staphylococcus aureus* (MRSA) and coagulase-negative staphylococci in both pediatric and adult patients (3, 4). The increasing use of vancomycin in pediatric intensive care unit (PICU) settings correlates with the growing incidence of MRSA and other resistant strains of gram-positive bacteria, underscoring its significance (5-9). To ensure optimal vancomycin use and mitigate antimicrobial resistance, it is essential to determine targeted serum concentrations through pharmacokinetic evaluations. Therefore, therapeutic drug monitoring is recommended to optimize vancomycin dosage and achieve the desired area under the curve over 24 hours (AUC_24_) (10, 11).

Pharmacokinetic parameters, such as volume of distribution and drug clearance, vary notably between the pediatric population and adults (2). Additionally, alterations in plasma protein binding, liver blood flow, hepatic metabolism, and renal clearance in critically ill patients may lead to changes in drug concentrations (12-14). However, data on vancomycin dosing and monitoring in the pediatric population are limited and contentious (15).

Recognizing the complexities of pediatric pharmacokinetics and the controversial data surrounding vancomycin administration in this population, the Infectious Diseases Society of America (IDSA) recommends that the ratio of AUC_24_ to the minimum inhibitory concentration of vancomycin (AUC_24_/MIC) should be ≥ 400 mg.h/L as the primary treatment goal. Moreover, vancomycin should be administered at dosages of 60 to 80 mg/day at 6- or 8-hour intervals to achieve this goal in pediatric patients (15). However, more than fifty percent of pediatric patients receiving routine doses of intermittent infusion of vancomycin (IIV) fail to reach the desired level (1, 4, 16-18). Continuous infusions of vancomycin (CIV) is proposed as one method to achieve the desired AUC_24_/MIC. Continuous infusions of vancomycin delivers the desired serum concentration faster than the IIV method, reduces subtherapeutic episodes, and may decrease drug toxicity (1, 4, 19).

In the quest for optimal treatment strategies for pediatric patients, CIV has emerged as an alternative to IIV. 

## 2. Objectives 

This clinical trial aims to compare these two methods based on the assessment of pharmacokinetic findings, including the achievement of target concentrations and AUC_24_, along with the evaluation of adverse reactions.

## 3. Methods

### 3.1. Study Setting

This double-blind, randomized, controlled clinical trial was conducted in the PICU at Mofid Children's Hospital, a tertiary referral center in Tehran, Iran. The research commenced in August 2022 and concluded in March 2023. Ethical approval was granted by the Ethics Committee of the Schools of Pharmacy, Nursing, and Midwifery at Shahid Beheshti University of Medical Sciences, as evidenced by the approval identifier IR.SBMU.PHARMACY.REC.1400.293. The trial protocol was registered with the Iranian Registry of Clinical Trials, with the registration number IRCT20120415009475N12. Standardized procedures were employed to secure written informed consent from the legal guardians, considering the vulnerable pediatric population involved in the study. The CONSORT 2010 statement was utilized to standardize the methodology used for this article. Therefore, the CONSORT checklist is available in the supplementary section.

### 3.2. Study Population

The inclusion criteria for the study population included pediatric patients receiving vancomycin treatment for proven or suspicious MRSA infections, aged between 2 months and 15 years, and weighing less than 67 kg, in line with the maximum dosing recommendation of 1000 mg. Additionally, inclusion required a glomerular filtration rate (GFR) ≥ 50 mL/min/1.73 m^2^, calculated using the Schwartz (Jaffe) equation, and availability of central venous access.

Conversely, the exclusion criteria aimed to exclude patients with a documented type 1 hypersensitivity reaction to vancomycin, those who had received vancomycin within the 72 hours preceding the study, individuals with a creatinine clearance < 50 mL/min/1.73 m^2^, and those undergoing renal replacement therapy, extracorporeal membrane oxygenation, or plasma apheresis. Additionally, the results did not include patients who had received vancomycin for a duration shorter than 72 hours or those who declined further participation and withdrew consent.

### 3.3. Randomization and Blinding

The trial employed a randomization protocol wherein patients were assigned to either the CIV or IIV group at a 1:1 ratio. This randomization utilized a computer-generated sequence with blocks of four to ensure balanced allocation. The randomization sequence was created by an independent statistician and implemented through a centralized randomization system managed by Sealed Envelope Ltd., which provided allocation concealment. The study adhered to a double-blinding methodology. Participants, their legal guardians, the data collectors, and the outcome assessors were all blinded to the treatment assignments. The pharmacy unit provided infusion bags, identical for both groups, via a concealment protocol under the supervision of independent third-party staff and a pharmacist. Emergency unblinding was permissible under strict protocols and could be conducted only by an independent adjudicator when the clinical management of a participant needed knowledge of the assigned treatment. At the study's conclusion, a blinding assessment was conducted to verify the integrity of the blinding process.

### 3.4. Data Collection

Baseline and clinical data were systematically compiled using a checklist specifically designed and validated by the researchers for this study. This checklist was refined to ensure consistency with established benchmarks, ensuring its reliability and comprehensiveness. The data collected included patient demographic information (age, gender, weight, height), medical history, underlying disease conditions, length of hospital and ICU stay, in-hospital vital status, and need for mechanical ventilation. An extensive range of clinical parameters were gathered from health records, including the administration of nephrotoxic medications such as antibiotics (carbapenems, aminoglycosides, colistin, amphotericin B, piperacillin-tazobactam, acyclovir, and remdesivir), ciclosporin, tacrolimus, diuretics, and inotropic drugs. Adverse drug reactions, specifically nephrotoxicity, were determined based on the kidney disease improving global outcomes (KDIGO) guidelines, while hypersensitivity reactions were identified using standardized protocols (20). The data collectors were blinded to the participants' group assignments. Additionally, laboratory data pertinent to the study's aims, such as vancomycin levels, serum creatinine levels, urine output, GFR, albumin levels, and total protein levels, were extracted from the laboratory test results. The pediatric risk of mortality (PRISM) and pediatric sequential organ failure assessment (PSOFA) were scored at predetermined intervals by employing validated checklists (21, 22).

Temperature measurements throughout the study were conducted using non-contact infrared thermometers, with periodic cross-verification using mercury thermometers to ensure accuracy and consistency.

### 3.5. Intervention

For the IIV regimen, vancomycin, provided by EXIR Company, Tehran, Iran, was administered at a prescribed dose of 60 mg/kg/day, divided into four doses every six hours and infused over one hour, following the standard dosing protocol. In contrast, the CIV group received an initial loading dose of 15 mg/kg within the first hour, followed by a continuous infusion rate equivalent to a daily dose of 60 mg/kg over the subsequent 24-hour period. Vancomycin was administered via infusion pumps by nurses under the supervision of a clinical pharmacist. The nurses received training on drug administration and intravenous medication incompatibilities from the clinical pharmacist, based on data from the handbook on injectable drugs: ASHP's guide to IV compatibility and stability (19th edition). All patients in both groups received 12 mL/kg/day continuous infusion and 3 mL/kg every 6 hours as a placebo or vehicle for vancomycin; this volume was deducted from their daily fluid intake. Adherence to the treatment protocol was meticulously documented in the patient's health record and audited by an independent reviewer to ensure accurate execution of the dosing schedule.

### 3.6. Blood and Ultrafiltrate Sampling and Vancomycin Concentration Determination

Peripheral blood samples (1 mL each) were collected from all patients on the third day post-vancomycin initiation. The first sample was obtained thirty minutes before the vancomycin infusion as the trough, and the second was taken one and a half hours after the infusion commenced, corresponding to the expected timing for peak drug concentrations. In the CIV group, samples were collected as in the intermittent group for blinding and concealment purposes and to enhance accuracy. The samples were centrifuged, stored in a freezer at -40°C, and subsequently sent to the laboratory for analysis. Vancomycin concentrations were measured using the COBAS INTEGRA 400 system (Roche Diagnostics, Switzerland), previously calibrated and validated for this assay to ensure accuracy.

### 3.7. Outcomes

The AUC_24_, representing the 48 - 72 hour period from the start of vancomycin therapy, was calculated using pharmacokinetic equations based on the two separate vancomycin levels, ensuring robust pharmacokinetic analysis (23).

The incidence of adverse drug reactions (ADRs) and nephrotoxicity (increased creatinine level ≥ 1.5 times the baseline or ≥ 0.3 mg/dL) was monitored during treatment.

The primary outcome was the comparison of serum vancomycin concentration and AUC_24_ between the CIV and IIV groups. All laboratory personnel measuring these outcomes were blinded to patient group assignments to ensure objective assessment.

Changes in PSOFA scores, SCr levels, and body temperature were systematically assessed from day one (baseline) to the fifth day of the intervention, with additional SCr measurements taken on the final day of vancomycin treatment and one day post-intervention. These time points were selected based on their clinical outcomes and expected pharmacodynamic response times. The occurrence of ADRs and nephrotoxicity was closely monitored.

### 3.8. Sample Size Calculation

The sample size was estimated using data from a prior study, which revealed that the target serum concentration was 85% in the CIV group and 41% in the IIV group among infants (11). We evaluated the suitability of these rates for our population and concluded that they were applicable. To detect a significant difference at a 5% alpha level with 90% power and accounting for an anticipated 15% attrition rate, 34 subjects per group were necessary. This calculation was conducted using STATA version 14, incorporating an adjustment for multiple comparisons. Our total sample size thus reached 68 subjects (n = 68).

### 3.9. Statistical Analysis

The normality of the distributions of continuous variables was assessed using the Kolmogorov-Smirnov (K-S) test, complemented by visual inspection of histograms with overlaid normal density curves. Descriptive statistics for continuous variables are presented as means and standard deviations (SDs), while frequencies and percentages are presented for categorical variables. The Mann-Whitney U test was used to compare non-normally distributed continuous variables between groups, as it allows unbiased comparisons irrespective of the underlying distribution. Categorical variables were compared using Fisher's exact test or the chi-square test based on the expected cell frequencies.

An intention-to-treat analysis was conducted to ensure comprehensive inclusion, encompassing all participants initially allocated after randomization. Temporal trends and the intervention's impact on outcomes were evaluated using unadjusted and adjusted generalized estimating equations (GEEs) with an exchangeable correlation structure. Adjustments in the GEE models were made for predefined confounders to ensure an unbiased estimation of the intervention effect. The optimal model was identified by the smallest quasi-likelihood under the independence model criterion (QIC). All statistical analyses were performed using STATA software version 14, maintaining a significance threshold of 0.05 and reporting 95% confidence intervals (CIs) to ensure precise and reliable inferential statistics.

### 3.10. Pharmacokinetic Modeling

Pharmacokinetic parameter estimation and modeling were performed using the Monolix software 2023R1 package (Lixoft^©^) under an academic license. Initial population parameters were estimated using the stochastic approximation expectation-maximization (SAEM) algorithm, which effectively manages the intricacies of the data inherent in pediatric pharmacokinetic studies. The algorithm was applied to linearization and structural models suited for the administration method and for estimating the volume of distribution (V) and clearance (Cl).

Automatic covariate model building was conducted using the stochastic approximation for model building algorithm (SAMBA), with model selection based on the lowest corrected Bayesian information criterion (BICc). The AUC_24_ for the steady state was calculated using the formula AUC_24_ = Dose24/Cl, with dose24 defined as the total dose of vancomycin administered per kilogram over 24 hours and Cl representing the clearance rate in liters per hour per kilogram (liter/h/kg).

Covariate modeling was systematically executed using the SAMBA strategy to identify significant physiological predictors of pharmacokinetic variability, aiming to increase the precision of pharmacokinetic parameters and minimize potential confounding. The final pharmacokinetic model was selected based on the achievement of the lowest BICc, optimizing the trade-off between goodness-of-fit and model simplicity.

## 4. Results

**Figure 1.**
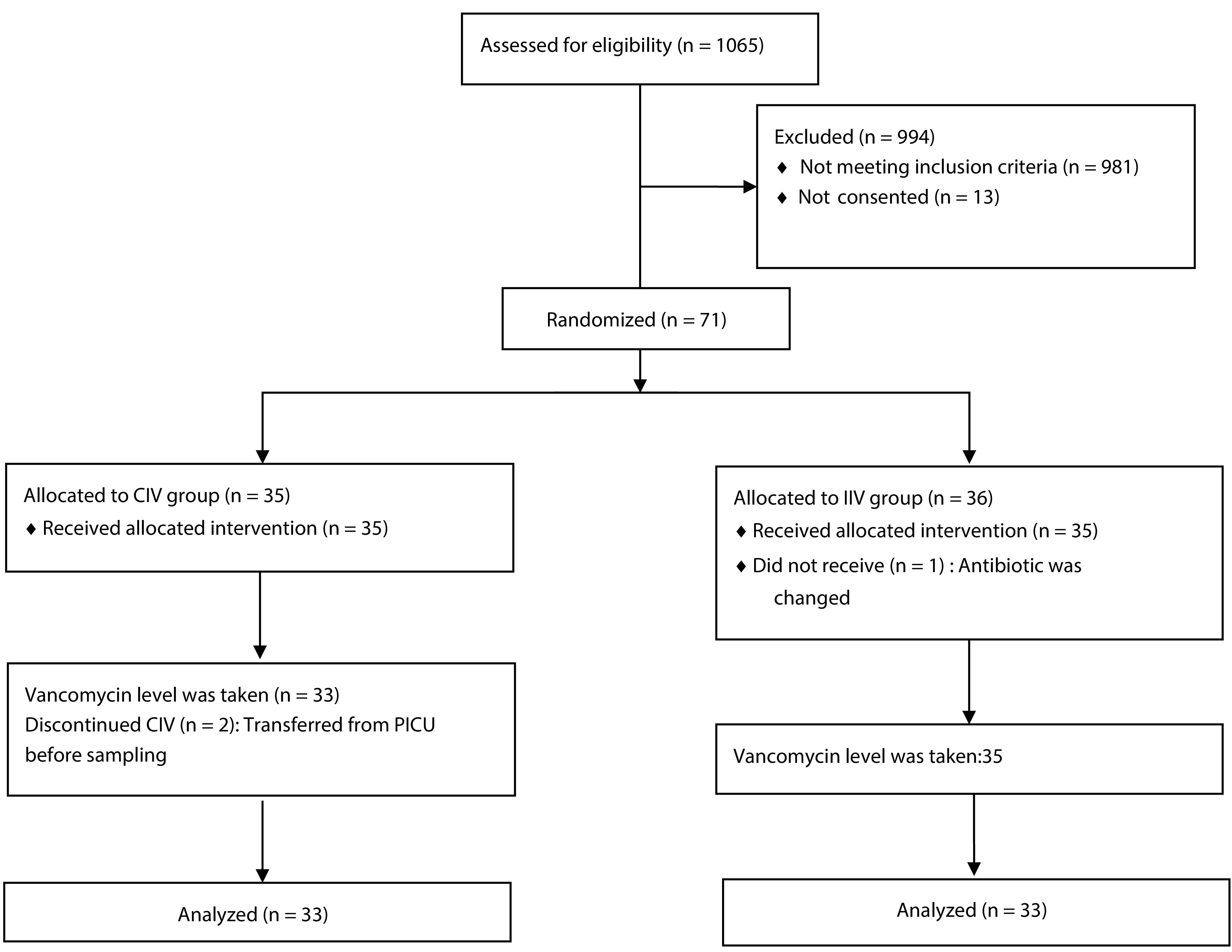
Study flow diagram. CIV, continuous infusions of vancomycin; IIV, intermittent infusions of vancomycin.

During the nine-month study period, 1065 patients were assessed for eligibility. Nine hundred and ninety four patients either did not meet the inclusion criteria or did not complete the consent form. Therefore, 71 patients were ultimately assigned to the study. The patients were randomly allocated, with 35 in the CIV group and 36 in the IIV group. Subsequently, 33 patients in the CIV group and 35 in the IIV group were included in the analysis (Figure 1). 

### 4.1. Characteristics of the Study Population

Demographics and baseline clinical parameters are summarized in Table 1. A comparative analysis revealed a significant discrepancy between the groups in the prescription of nephrotoxic and inotropic medications (P-value = 0.043), with a notably greater incidence in the CIV group (42.42%) than in the IIV group (17.14%, P-value = 0.022).

**Table 1.** Comparison of General Information on Critically Ill Pediatrics Between the Two Groups of Intervention ^a^

Variables	All Patients (n = 68, 100%)	CIV (n = 33, 48.53%)	IIV (n = 35, 51.47%)	P-Value
**General information**				
Age (mo)	38.96 ± 43.85	35.50 ± 41.75	42.22 ± 46.10	0.305
Age (y)				0.490
Younger than one year	26 (38.24)	14 (42.42)	12 (34.29)	
Older than one year	42 (61.76)	19 (57.58)	23 (65.71)	
Gender				0.038 ^b^
Female	25 (36.76)	8 (24.24)	17 (48.57)	
Male	43 (63.24)	25 (75.76)	18 (51.43)	
Underlying diseases				0.190
CNS disease	11 (16.18)	6 (18.18)	5 (14.29)	
Endocrine and metabolic syndrome	5 (7.35)	2 (6.06)	3 (8.57)	
Gastrointestinal diseases	15 (22.06)	10 (30.30)	5 (14.29)	
Blood disorders	5 (7.35)	0 (0.00)	5 (14.29)	
Others	11 (16.18)	6 (18.18)	5 (14.29)	
None	21 (30.88)	9 (27.27)	12 (34.29)	
Chief complaint (yes)				0.079
Seizure	9 (13.24)	7 (21.21)	2 (5.71)	
Loss of consciousness	4 (5.88)	4 (12.12)	0 (0.00)	0.050
Post-operation	22 (32.35)	12 (36.36)	10 (28.57)	0.492
Respiratory distress	34 (50.00)	11 (33.33)	23 (65.71)	0.008 ^b^
Others ^c^	10 (14.71)	5 (15.15)	5 (14.59)	0.920
**Hospitalization and follow-up during treatment**				
Vital status				
Alive discharged	47 (70.15)	20 (60.61)	27 (79.41)	0.093
Died	20 (29.85)	13 (39.39)	7 (20.59)	
Mechanical ventilation (yes)	44 (64.71)	25 (75.76)	19 (54.29)	0.064
Ventilation time (day)	10.25 ± 8.46	10.26 ± 8.29	10.23 ± 8.87	0.755
Length of ICU stay (day)	22.30 ± 24.94	22.54 ± 14.10	22.17 ± 32.23	0.168
Length of hospital stay (day)	30.14 ± 30.76	27.63 ± 15.12	32.51 ± 40.46	0.457
**Clinical characteristics**				
Height (cm)	88.54 ± 26.18	86.21 ± 24.30	90.74 ± 28.01	0.407
Weigh (kg)	13.19 ± 10.50	13.12 ± 11.77	13.25 ± 9.32	0.491
Pediatric Risk of Mortality (PRISM) score	6.91 ± 4.24	7.72 ± 4.09	6.14 ± 4.29	0.097

Abbreviations: CIV, continuous infusions of vancomycin; IIV, intermittent infusions of vancomycin; CNS, central nervous system; PRISM, pediatric risk of mortality.

^a^ Values are expressed as No. (%) or mean ± SD.

^b^ Statistically significant at P-value < 0.05.

^c^ Such as respiratory, cardiovascular, musculoskeletal, or neuromuscular disorders.

### 4.2. Serum Level and AUC24 of Vancomycin

A total of 136 blood samples were collected from 68 patients within 48 to 72 hours of starting vancomycin treatment. The results are presented in Table 2. 

**Table 2.** Comparison of Clinical Information of Critically Ill Pediatrics Between Two Groups of Intervention ^a^

Variables	All Patients (n = 68, 100%)	CIV (n = 33, 48.53%)	IIV (n = 35, 51.47%)	P-Value
**Vancomycin related information**				
Serum level 1 or trough level at steady state (mg/dL) with dose of 60 mg/kg/day	14.63 ± 9.39	19.51 ± 9.74	10.03 ± 6.30	< 0.001 ^b^
Area under the serum drug concentration-versus-time curve mg.h/L (AUC_24_)	474.05 ± 234.14	608.52 ± 248.74	347.26 ± 125.31	< 0.001 ^b^
< 400	33 (48.53)	6 (18.18)	27 (77.14)	
400 - 600 (appropriate range)	21 (30.88)	15 (45.45)	6 (17.14)	
600 - 800	8 (11.76)	6 (18.18)	2 (5.71)	
> 800	6 (8.82)	6 (18.18)	0 (0.00)	
Reaction during drug injection (yes)	1 (1.47)	1 (3.03)	0 (0.00)	0.299
**Laboratory findings on the third day of treatment**				
Albumin (g/dL)	3.42 ± 0.52	3.41 ± 0.43	3.43 ± 0.64	0.700
Total protein (g/dL)	5.36 ± 0.95	5.33 ± 0.97	5.41 ± 0.95	0.980
Urine output (mL/kg/h)	3.55 ± 1.41	3.62 ± 1.62	3.48 ± 1.20	0.970
Glomerular filtration rate (mL/min)				
**In-hospital medication**	96.02 ± 39.52	94.12 ± 40.58	97.80 ± 39.006	0.446
Total number of drugs prescription	2.94 ± 1.46	3.30 ± 1.51	2.60 ± 1.35	0.043 ^b^
Antibiotics (yes)	68 (100.00)	33 (100.00)	35 (100.00)	N/A
Total number of antibiotic prescriptions	1.92 ± 0.93	2.00 ± 0.93	1.85 ± 0.94	0.434
Inotropic drugs (yes)	20 (29.41)	14 (42.42)	6 (17.14)	0.022 ^b^
Total number of inotropic drugs prescription	0.47 ± 0.78	0.72 ± 0.91	0.22 ± 0.54	0.013 ^b^
Diuretics (yes)	30 (44.12)	18 (54.55)	12(34.29)	0.093
Angiotensin-converting enzyme inhibitors (yes)	7 (10.29)	1 (3.03)	6 (17.14)	0.056

Abbreviations: CIV, continuous infusions of vancomycin; IIV, intermittent infusions of vancomycin; N/A, not applicable.

^a^ Values are expressed as No. (%) or mean ± SD.

^b^ Statistically significant at P-value < 0.05.

### 4.3. PSOFA, Serum Creatinine, and Body Temperature

Statistically significant temporal trends were observed in PSOFA scores and SCr levels, with a significant interaction effect between time and treatment group (P < 0.001) (Appendices 1, 4, and 5). No significant changes or interaction effects were noted in body temperature measurements (P > 0.05) (Appendix 6).

### 4.4. Adjustment for Confounding Factors

According to the univariate regression model of the GEE (Appendix 2), a significant association was found between the type of intervention and the changes in PSOFA score in children. Specifically, the mean PSOFA score was lower in the IIV group than in the CIV group (β = -1.84, 95% CI = -3.56, -0.13; P-value = 0.035).

After considering the effect of the other variables under investigation and possible confounders according to the results of the multivariable linear GEE regression model, no statistically significant associations were found between the intervention methods and the mean changes in the three considered outcomes (PSOFA, serum creatinine, body temperature) during the study (p value > 0.05).

### 4.5. Serum Level and AUC24 of Vancomycin Determined by Pharmacokinetic Modeling

**Figure 2.**
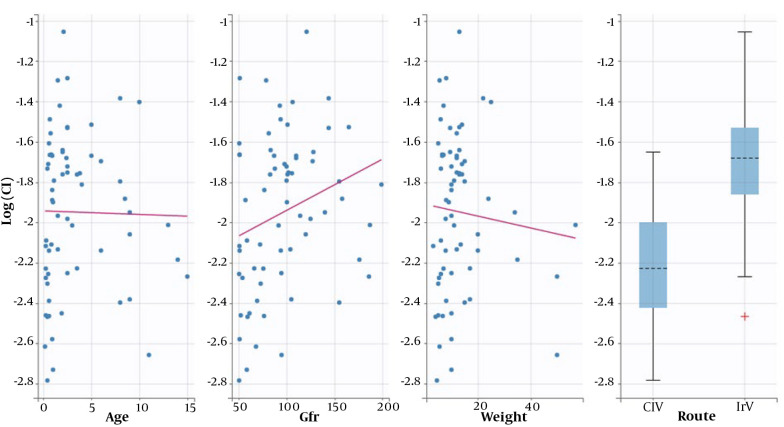
Goodness-of-fit plots: The effect of age, GFR, weight, and method of administration (CIV vs. IIV) on the model and method of administration was proposed to affect clearance. CIV, continuous infusions of vancomycin; IIV, intermittent infusions of vancomycin; GFR, glomerular filtration rate.

Following initial model building, a one-compartmental structural model was determined to be the most suitable for describing the population pharmacokinetic parameters of Cl and V. The SAMBA method was utilized for automatic covariate model building to assess the effect of age, weight, GFR, and method of administration (CIV vs. IIV) on the model. The method of administration was found to affect the Cl concentration (Appendix 3). The proposed population parameters after SAEM estimation are presented in Table 3. Model fitness was evaluated using the prediction distribution versus time (Appendix 7). The final model revealed no significant correlations between the method of administration and weight, age, or GFR (Figure 2). 

**Table 3.** Estimates of Pharmacokinetic Parameters of the Final Model ^a^

	Value	CV (%)	Linearization	
			SE	RSE (%)
**Fixed effects**				
V-pop	0.88		0.096	10.8
Cl-pop	0.11		0.0074	7.04
Beta-Cl-route-IIV	0.48		0.097	20.4
**Standard deviation of the random effects**				
Omega-V	0.33	33.96	0.14	43.8
Omega-Cl	0.33	34.31	0.04	11.9
**Error model parameters**				
B	0.28	0.032	11.5	

Abbreviations: CIV, continuous infusions of vancomycin; IIV, intermittent infusions of vancomycin; RSE, relative standard error; SE, standard errors; %CV, coefficient of variation; V, volume of distribution (L/kg); CL, clearance (L/h/kg), omega the standard deviation.

^a^ The final individual model was built based on the following equations: Log(V) = log(V-pop) + eta-V and log (Cl) = log (Cl-pop) + beta-Cl-route-IIV*[route = IIV] + eta-Cl.

To assess the PK/PD target achievement of each administration method, AUC_24_ (AUC_48_72_ assumed to be steady state) was calculated as 587.7 ± 184.4 and 361.9 ± 113.2 mg.h/L for the CIV and IIV methods, respectively (P-value < 0.05). The goal of an AUC_24_ ≥ 400 mg.h/L was achieved in 87% vs. 23% of patients in the CIV and IIV groups, respectively.

### 4.6. Adverse Events

Nephrotoxicity was reported in two patients, one from each treatment group. Standardized monitoring procedures for ADRs were applied across both groups to ensure uniform detection and reporting. One patient in the CIV group experienced an infusion reaction while receiving the loading dose, which was successfully managed with an antihistamine agent and an adjusted infusion rate, with no subsequent recurrence noted.

## 5. Discussion

This trial represents the first study directly comparing the achievement of the recommended AUC_24_ using two administration methods, IIV and CIV, in pediatric patients aged 2 months to 15 years admitted to the ICU. The results showed that the AUC_24_ in the CIV group was greater than that in the IIV group. The pharmacokinetics of vancomycin vary in the pediatric population (8, 19, 20), and there are several controversies surrounding its dosing and monitoring in this population based on age and underlying medical conditions (21, 22). Our study demonstrated that the CIV method has significantly more favorable results than the IIV method.

Several studies have reported vancomycin serum concentrations of 60 mg/kg/day administered intermittently (10, 24). In critically ill pediatric patients, one study revealed that 69% had serum drug concentrations below the desired therapeutic range (25). Conversely, Hoegy et al. reported that 60% of pediatric patients achieved the target serum level of 14 to 21 mg/L with continuous infusion (26). Furthermore, CIV with a loading dose has been associated with more rapid attainment of target serum levels in neonates and children, with more than 60% of neonates and children achieving these levels more swiftly than with intermittent infusion (27-29). Based on these studies, the loading dose was implemented in this study. In McKamy et al.'s study, when the treatment method for patients with an average serum concentration of 9.2 ± 4.6 mg/L was changed to CIV, 80% of the patients reached the therapeutic level of 19.1 ± 3.05 mg/L (30). While studies have not conclusively explained why serum levels are greater with continuous infusion, our data suggest that a decrease in vancomycin clearance with the CIV method could also contribute to this difference.

Additionally, the mean AUC_24_ of vancomycin in the CIV group was almost twofold greater than that in the IIV group. Notably, 77% of the patients in the IIV group and only 18% of those in the CIV group had AUC_24_ values less than 400 mg.h/L. Dose adjustment was promptly performed for the six CIV patients with AUC_24_ values above 800 mg.h/L. The literature lacks studies comparing the AUC_24_ of vancomycin in pediatric patients treated with IIV or CIV, although separate evaluations of each method exist. For instance, Mali et al. reported an estimated AUC_24_ of vancomycin in the IIV method (dose: 60 mg/kg/day) of 372.44 ± 153.82 mg.h/L; however, the proportion of patients with AUC_24_ values less than 400 mg.h/L was not specified (10). Fewer studies have reported the AUC_24_ of the CIV method (31, 32), with one showing an average AUC_24_ of 355 mg.h/L (range = 261 - 1001) (12).

The relationship between the administration method and clinical outcomes was not significantly different. Confounding factors such as age, gender, PRISM score, coprescribed antibiotics, and length of stay were examined, but no significant differences were observed between the two groups. However, the small sample size limits the power of these findings.

Our study revealed no significant difference in mortality between the CIV and IIV groups, which aligns with the findings of a systematic review of adults reporting a relative risk of 0.94 (95% CI = 0.72 - 1.25) (3). Nonetheless, a retrospective study suggested a reduction in mortality from pneumonia caused by MRSA when treated with CIV (33), indicating that the effects of administration methods on mortality may vary across patient populations and disease etiologies. The incidence of nephrotoxicity was similar in both groups, in contrast with the findings of several studies reporting a significantly lower incidence of nephrotoxicity with CIV (34); however, a meta-analysis reported that this reduction in nephrotoxicity risk was not significant (risk ratio = 0.799, 95% CI = 0.523 - 1.220; P = 0.299) (35). Therefore, while it is not definitive that CIV reduces nephrotoxicity risk, the available evidence does not suggest that CIV is associated with a greater risk than IIV is. Our study did not observe drug incompatibilities or infusion-related adverse effects due to CIV. However, RCTs with larger populations are suggested to substantiate findings concerning mortality, ADRs, and nephrotoxicity.

Pharmacokinetic parameters were calculated using computer-based pharmacokinetic modeling and traditional formulas for comparison. Computer modeling showed that vancomycin clearance was lower in patients receiving continuous infusion in our population, consistent with the results of previous studies and may explain the difference between studies of intermittent and continuous methods. Furthermore, computer modeling was in line with the traditional formula, demonstrating the robustness of the results.

This study has several limitations. The paucity of positive cultures limits microbiological assessment and, consequently, the evaluation of MIC values. Additionally, the PICU setting precluded comprehensive audiology assessments, inhibiting our ability to comment on the ototoxic potential of vancomycin. Future studies should incorporate thorough microbiological evaluations, including AUC_24_/MIC ratios and, where feasible, auditory monitoring. Moreover, given the diversity within pediatric populations, tailored population pharmacokinetic models are necessary to establish more definitive dosing guidelines for pediatric vancomycin administration. Future studies should focus on elucidating the specific pharmacokinetic mechanisms responsible for the observed increase in drug levels with CIV and on assessing the potential benefits on clinical outcomes across diverse pediatric subpopulations, including those with a low estimated glomerular filtration rate (eGFR).

### 5.1. Conclusions

In conclusion, this RCT demonstrated that continuous vancomycin infusion achieves a higher AUC_24_ compared to intermittent vancomycin infusion, with a greater success rate in attaining an AUC_24_ ≥ 400 mg.h/L. This finding is particularly beneficial for safely achieving therapeutic vancomycin levels in PICU patients.

ijpr-23-1-145933-s001.pdf

## Data Availability

The dataset presented in the study is available on request from the corresponding author during submission or after its publication. The data are not publicly available due to internal policy.

## Clinical Trial Registration Code:

This study is registered with the Iranian Registry of Clinical Trials (IRCT) under the code IRCT20120415009475N12.
