# Supplementary material for: Comparison of the Areas Under the Curve of Vancomycin Continuous vs. Intermittent Infusion in Critically Ill Pediatrics: A Randomized Clinical Trial
Source: Iran J Pharm Res. 2024 Apr 28;23(1):e145933. doi: 10.5812/ijpr-145933 (PMC11302439; doi:10.5812/ijpr-145933)
Supplement: ijpr-23-1-145933-s001.pdf [file ijpr-23-1-145933-s001.pdf]

Appendix 1. Changes in the mean of Pediatric Sequential Organ Failure Assessment (PSOFA score), mean serum creatinine and mean of body temperature among critically ill pediatrics between two methods of vancomycin infusion based on the GEE method

Factors	Groups	Day 1	Day 2	Day 3	Day 4	Day 5	Last day of intervention	after intervention cessation	P_value time effect	P_value time × groups	Comparison of time groups [1]
PSOFA	CIV	8.30 ± 4.03	8.00 ± 3.90	7.06 ± 3.86	6.54 ± 3.83	6.60 ± 4.28	-	-	0.017*	<0.001*	T1/T2* T1/T3* T1/T4* T1/T5*
	IIV	6.62 ± 4.17	5.74 ± 3.77	4.88 ± 4.04	4.71 ± 4.06	4.76 ± 4.02	-	-	0.001*		T1/T3* T1/T4* T1/T5*
	Total	7.44 ± 4.16	6.83 ± 3.97	5.94 ± 4.07	5.57 ± 4.03	5.59 ± 4.21	-	-	<0.001*	-	T1/T2* T1/T3* T1/T4* T1/T5*
Serum creatinine (µmol/L)	CIV	0.52 ± 0.15	0.55± 0.15	0.53± 0.14	0.50± 0.17	0.49± 0.12	0.55± 0.17	0.51± 0.15	0.093	<0.001*	Non-significant
	IIV	0.54 ± 0.08	0.60± 0.65	0.47±0.08	0.49± 0.08	0.46±0.12	0.52 ± 0.18	0.55± 0.26	<0.001		T1/T3* T1/T4* T1/T5*
	Total	0.53 ± 0.12	0.57± 0.45	0.50± 0.12	0.49 ± 0.14	0.47 ± 0.12	0.53 ± 0.18	0.53± 0.21	0.005*	-	T1/T3* T1/T4* T1/T5*
Temperature (°C)	CIV	37.53 ± 0.75	37.59 ± 0.94	37.40 ± 0.72	37.45 ± 0.65	37.38 ± 0.57	-	-	0.235	0.225	Non-significant
	IIV	37.60 ± 0.77	37.63 ± 0.73	37.43 ± 0.59	37.41 ± 0.49	37.46 ± 0.58	-	-	0.208		Non-significant
	Total	37.57 ± 0.76	37.61 ± 0.84	37.42 ± 0.65	37.43 ± 0.57	37.42 ± 0.58	-	-	0.040*	-	Non-significant

Values described as mean ± standard deviation,
* Statistically significant, P_value < 0.05 based on the linear Generalized Estimation Equation (GEE) method
[1] each visit time compared with visit time 1
CIV Continuous Infusions of Vancomycin, *IIV* Intermittent Infusions of Vancomycin

Appendix 2. Results of the linear generalized estimating equation about the effect of intervention on mean changes of serum creatinine level, PSOFA and body temperature during the study

Factors	Groups	Model 1<br>Crude ß[1], 95% CI	P_value	Model 2<br>Adjusted ß, 95% CI	P_value
Serum creatinine (µmol/L)	CIV	Reference	-	Reference	-
	IIV	-0.002 (-0.05, 0.05)	0.933	0.01 (-0.04, 0.07)	0.603
PSOFA	CIV	Reference	-	Reference	-
	IIV	-1.84 (-3.56, -0.13)	0.035*	-0.58 (-1.77, 0.59)	0.534
Temperature (°C)	CIV	Reference	-	Reference	-
	IIV	0.02 (-0.22, 0.28)	0.831	0.09 (-0.20, 0.37)	0.539

[*] Statistically significant, P_value < 0.05

[1]Coefficient (ß)

Model 1: Intercept groups

Model 2: Intercept gender, age, groups, inotrope drugs, total number of prescription drugs during hospitalization, PRISM score and length of hospital stay

CIV Continuous Infusions of Vancomycin, *IIV* Intermittent Infusions of Vancomycin, *PSOFA* Pediatric Sequential Organ Failure Assessment, *CI* Confidence Interval

Appendix 3. Initial model building to describe the population pharmacokinetic parameters of clearance (Cl) and volume of distribution (V), one compartmental structural model was the best model

Structural model	one compartment infusion	one compartment infusion	two compartment infusion
Individual model	V, Cl	V, Cl	Cl, V1, Q, V2
Used covariates	administration method	-	-
-2*LL (Lin)	873.34	895.94	916.64
BICc (Lin)	900.49	923.76	962.55

CIV Continuous Infusions of Vancomycin, *IIV* Intermittent Infusions of Vancomycin, *-2*LL* (Lin) -2*log-likelihood computed by linearization, *BICc* (Lin) corrected the Bayesian Information Criteria based on the log-likelihood computed by linearization

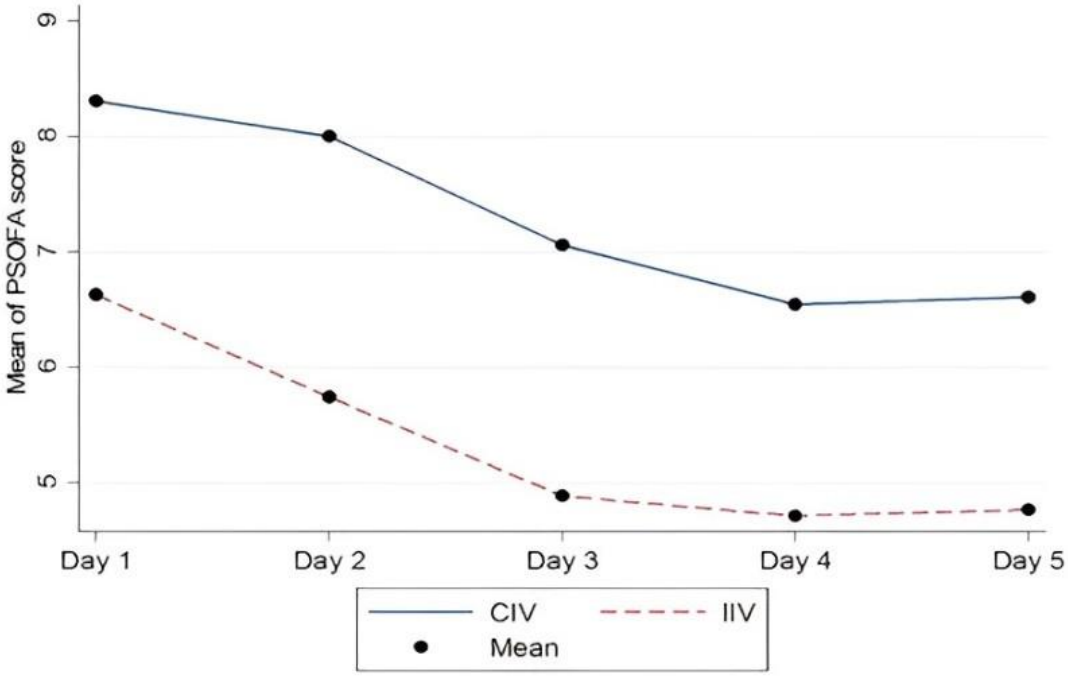

Appendix 4. Changes in the mean of Pediatric Sequential Organ Failure Assessment (PSOFA score) of critically ill pediatrics between two methods of vancomycin infusion during study time. *CIV* Continuous Infusions of Vancomycin, *IIV* Intermittent Infusions of Vancomycin

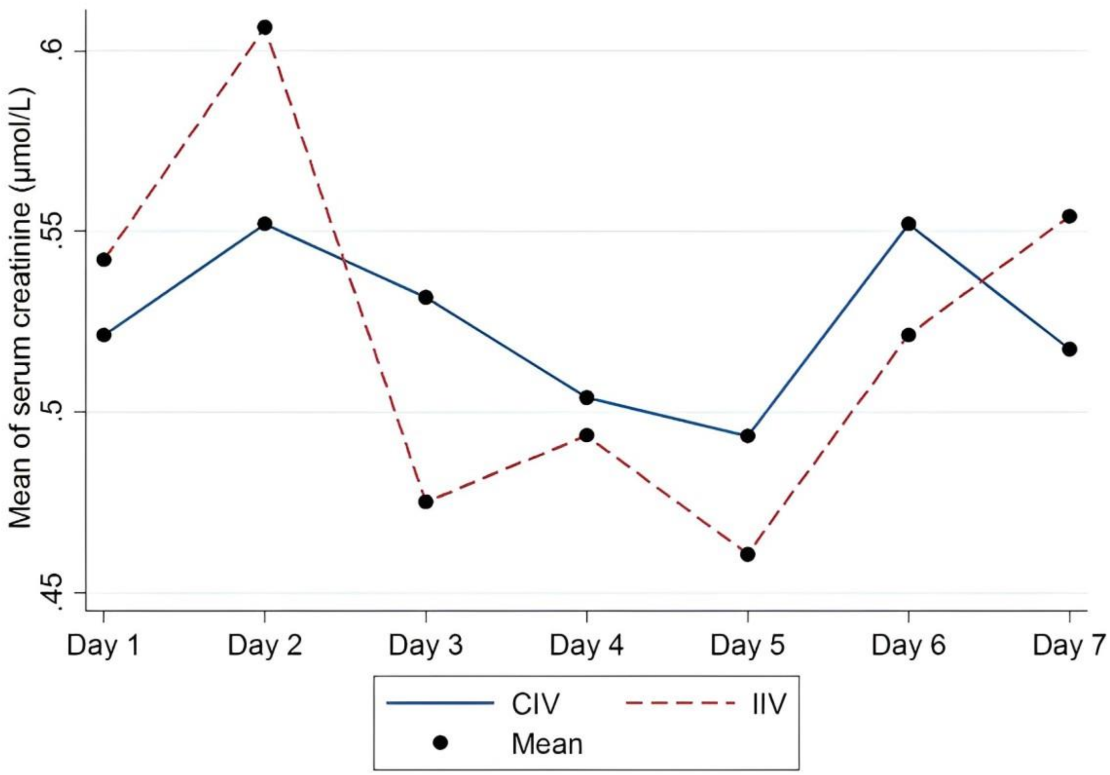

Appendix 5. Changes in mean of serum creatinine of critically ill pediatrics between two methods of vancomycin infusion during study time. *CIV* Continuous Infusions of Vancomycin, *IIV* Intermittent Infusions of Vancomycin

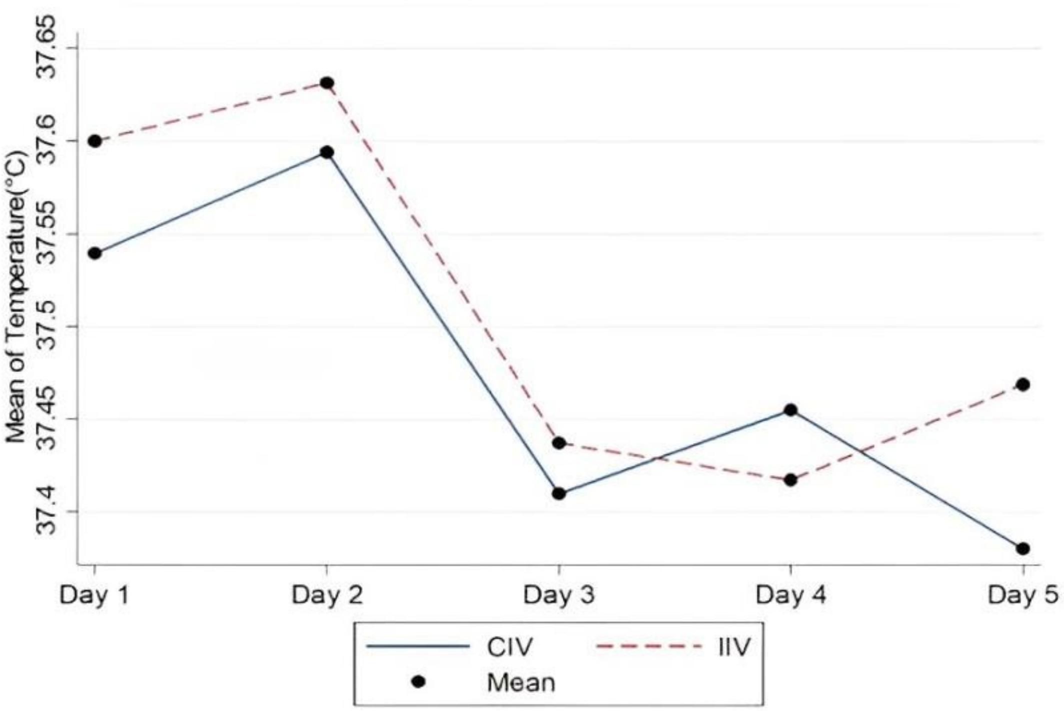

Appendix 6. Changes in mean body temperature of critically ill pediatrics between two methods of vancomycin infusion during study time. *CIV* Continuous Infusions of Vancomycin, *IIV* Intermittent Infusions of Vancomycin

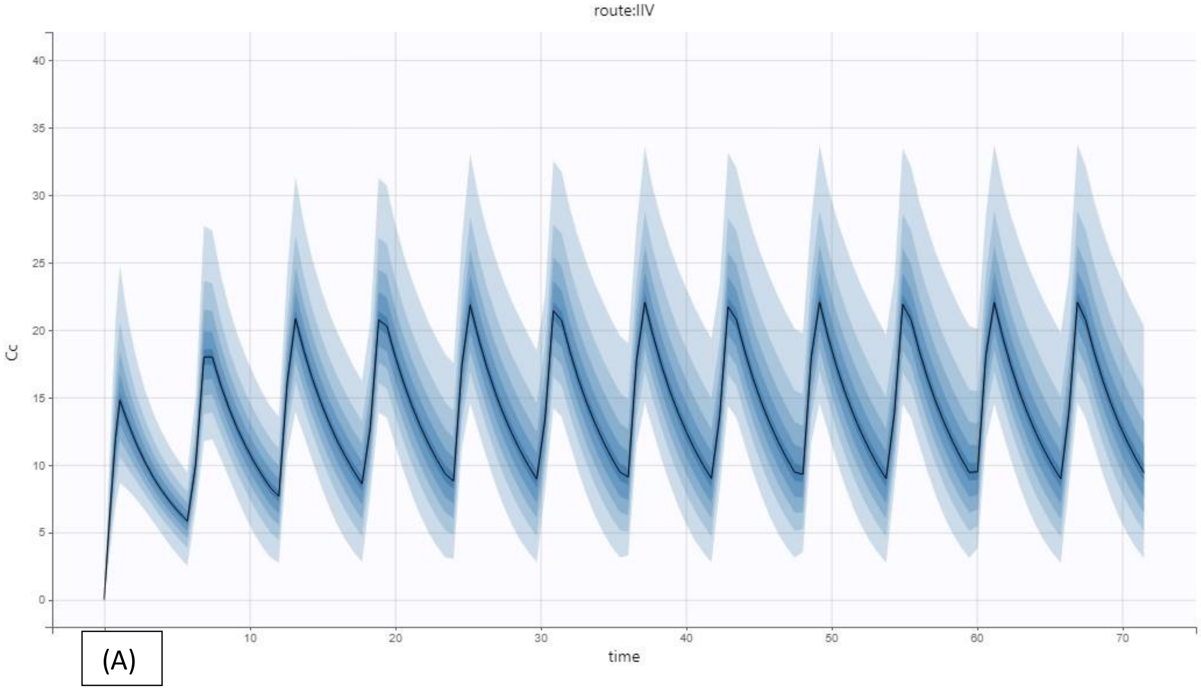

(A)

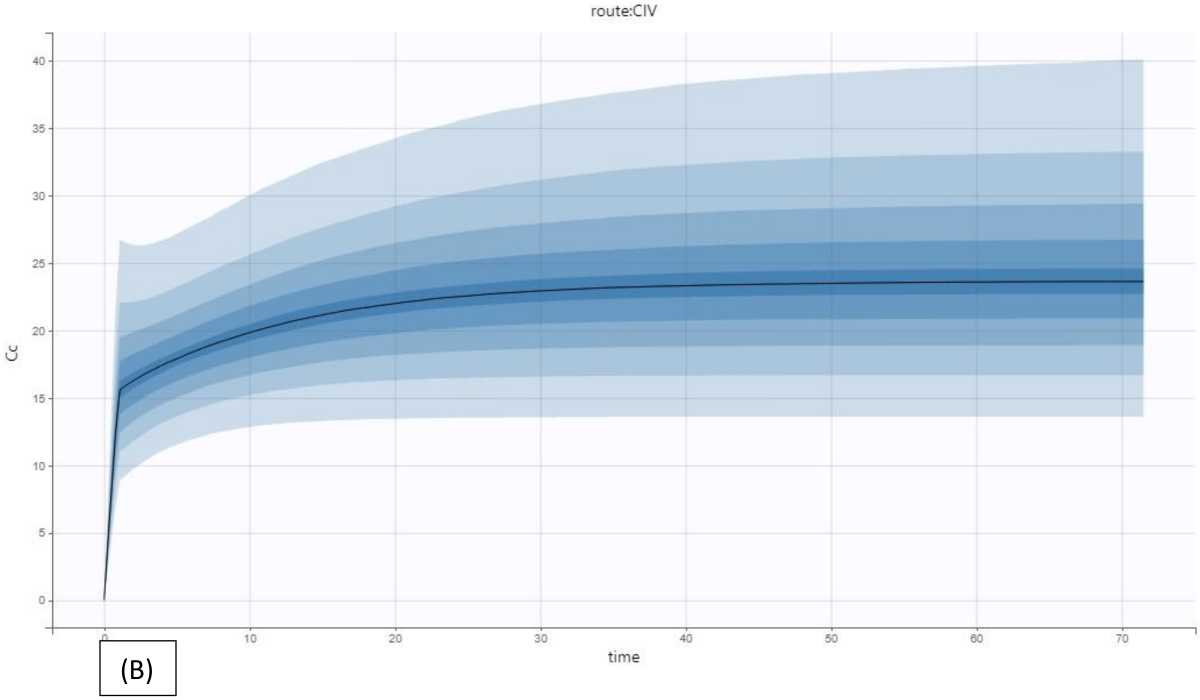

(B)

Appendix 7. Predicted-corrected visual predictive check for vancomycin concentration versus time A) intermittent infusion of vancomycin method (IIV) B) continuous infusion of vancomycin method (CIV)
